# Four year evaluation of a parent advisory group to support a research program for knowledge translation in child health

**DOI:** 10.1186/s40900-024-00547-5

**Published:** 2024-01-28

**Authors:** Lisa Hartling, Sarah A. Elliott, Annie Mabbott, Julie Leung, Kathleen Shearer, Chrissy Smith, Shannon D. Scott

**Affiliations:** 1 Department of Pediatrics, Faculty of Medicine and Dentistry, University of Alberta, Edmonton, AB Canada; 2 Faculty of Nursing, University of Alberta, Edmonton, AB Canada; 3 Pediatric Parent Advisory Group, University of Alberta, Edmonton, AB Canada

**Keywords:** Parents, Advisory group, Patient engagement, Patient-oriented research, Child health, Knowledge translation, Knowledge mobilization, Evaluation

## Abstract

**Background:**

In 2016, we developed a pediatric parent advisory group to inform our research program which creates innovative knowledge translation (KT) tools for parents on priority topics related to acute childhood illness. We implemented a mixed methods strategy to evaluate the experiences of group members. The purpose of this paper is to present the findings from parent evaluations over four years and to discuss our experiences collaborating with the group over a multi-year period.

**Methods:**

We conducted year-end surveys and interviews of group members to understand parents’ perceptions of their experiences, group management, researcher interaction, and other outcomes of advisory group participation from 2018 to 2021. We applied a mixed methods approach, collecting and analyzing both quantitative (survey) and qualitative (survey/interview) data. Survey data were analyzed by term using descriptive statistics (i.e., frequencies, percentages). Open-ended survey responses were analyzed by conventional content analysis. Interview data were analysed thematically.

**Results:**

Year-end survey response rates and interview participation varied over the years. Responses to evaluation questions were generally positive and most improved over time. Results prompted changes to improve P-PAG operations, such as changes to location of meetings, communications about the group’s purpose, offering sufficient context for discussion items, and providing feedback about how members’ input was used. Themes identified from the qualitative data related to the importance of certain aspects of group functioning, positive views of the group’s current management, and potential areas for improvement. Parents regularly expressed a desire for more diversity in the group’s membership and an interest in hearing more about how the research program’s activities fit into the broader healthcare system and their impacts on health outcomes.

**Conclusions:**

Our experience in establishing, managing, and evaluating a parent advisory group over many years has resulted in valuable insights regarding patient engagement in health research and sustaining an advisory group over time. We have learned that an intentional and iterative approach with regular evaluations and responsive changes has been essential for fostering meaningful engagement. Significant resources are required to maintain the group; in turn, the group has made substantial and diverse contributions to the research program and its outputs.

**Supplementary Information:**

The online version contains supplementary material available at 10.1186/s40900-024-00547-5.

## Abstract (plain language summary)

We developed a parent advisory group in 2016 to support our research program in knowledge translation (KT, i.e., sharing research in accessible ways to inform decision-making) for child health. The purpose of the group is to involve parents in co-developing, evaluating, and sharing KT tools (e.g., videos, infographics). The group has also worked with researchers to inform methods and provide input on research projects and products. The group has been running for seven years and has involved different types of evaluations, including parent surveys and interviews. Parents had generally positive views of their experience with the group (including the group’s management, interactions with researchers, etc.) and their responses mostly improved over the years. Based on parents’ feedback, we made changes to improve the group’s operations, such as changing the frequency and location of meetings, regular communications about the group’s purpose, offering more context and time for individual discussion items, and providing feedback to parents about how their input was used. Parents regularly expressed a desire for more diversity in the group’s membership. They also expressed an interest in hearing more about how the research program fit into the broader healthcare system and impacts on health outcomes. We have learned that it takes a large amount of time, effort, and funding to run the group; however, the parent contributions have been valuable and wide-reaching. We feel that the evaluations and responsive changes to the group over time have been essential to sustain and foster meaningful engagement and achieve the group’s objectives.

**Supplementary Information:**

The online version contains supplementary material available at 10.1186/s40900-024-00547-5.

## Background

Over the last two decades there has been a growing effort to involve patients and their advocates in health research [1]. The recognized benefits of patient involvement in research include increasing the relevance of research outputs, optimizing knowledge translation (KT, i.e., communication and uptake of evidence-based research by the intended knowledge users), enhancing research processes, and ultimately improving health outcomes. By sharing their lived experiences, patients and families can help shape the nature, conduct, and focus of health research [2]. Major funding organizations (e.g., Canadian Institutes of Health Research, Patient-Centered Outcomes Research Institute in the United States) recognize these benefits and support the involvement of patients and their advocates as active partners in research [3, 4].

We developed the Pediatric Parent Advisory Group (P-PAG) in 2016 to support our research program that focuses on KT in child health. Specifically, the purpose of the group is to involve parents in an advisory capacity to co-develop, evaluate, and disseminate innovative KT tools (e.g., whiteboard animation videos, interactive infographics) for parents and families on priority topics in acute childhood illness, and inform research strategies (e.g., recruitment processes, pilot testing data collection tools). Our KT tools integrate parents’ lived experience with evidence about various childhood illnesses to increase parent knowledge and support decision-making regarding their child’s health (e.g., when to seek emergency care, how to manage symptoms at home, etc.). To date we have developed more than 40 tools on 15 topics (e.g., bronchiolitis, asthma, fever, gastroenteritis, concussion, COVID-19) that are publicly available [5] and actively disseminated through various channels (e.g., health system, healthcare or community organizations, social media, etc.). Our research program is based at an academic institution. The leads of the research program are professors and also co-directors of a national centre of excellence in knowledge mobilization (trekk.ca) through which we interact with front-line healthcare providers to support evidence-based emergency care of children.

**Fig. 1:**
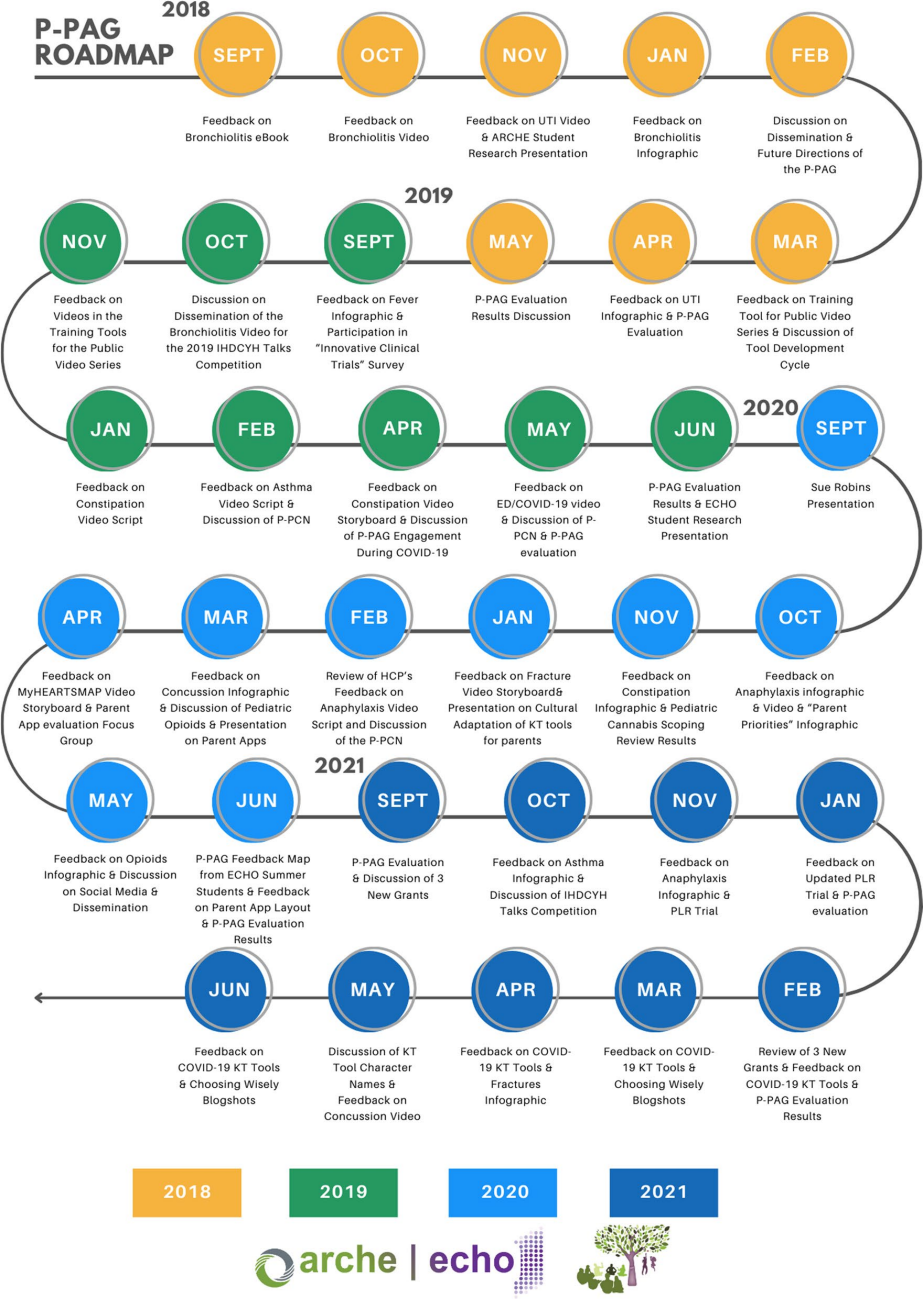
P-PAG road map of activities 2018–2021

Our KT tool development cycle involves multiple steps: topic prioritization; systematic reviews of existing literature; qualitative interviews with parents to identify experiences and information needs; developing prototypes of the KT tools; initial evaluation of prototypes by parents and healthcare providers or other content experts; refinement then usability testing of the tools; and dissemination [6]. At the center of our tool development process is the P-PAG that provides input on our processes and tools throughout the tool development and evaluation processes. The P-PAG meets regularly (six to nine times per year, between September and May or June) in-person or virtually, and regular communications (approximately twice per month) occur via email. A co-chairing system in which two parents take turns chairing meetings has been in place since 2018. Typically, we discuss two to three topics per meeting. Discussions are most often facilitated by one of the research staff who have training and experience in qualitative methods (e.g., leading focus groups, deliberative dialogue). When more than 8–10 parents attend a given meeting, we split into two groups to allow for easier discussion of specific items, then share a summary of discussions with the whole group; often the second group is facilitated by one of the parent chairs. On occasion another researcher presents at a meeting and on various occasions graduate students have presented their research to gather parent feedback. Figure 1 summarizes the topics discussed at each of the P-PAG meetings during the period under evaluation in this paper. At the end of each term some parents leave the group, therefore, we recruit new parents leading up to the start of each new term. Recruitment occurs by advertising through our networks, via social media, and posting flyers at public places (e.g., libraries, cafes). We seek parents, grandparents or legal guardians of children younger than 18 years who want to contribute to child health research, are willing to work collaboratively with a group, and are able to attend regular meetings [6]. Interested parents meet with a member of the research team to discuss details and together decide on suitability for membership, and how they can be involved in the program.

Despite an increasing interest in patient-oriented research (POR), there is a substantial gap in evidence that assesses the extent and impact of patient engagement in child health; moreover, there are very few published evaluations of patient engagement activities [7, 8]. Evaluation of engagement efforts are needed to understand what works and how to improve the process. We developed and implemented an a priori evaluation plan of the P-PAG to ensure that we were meeting our overarching goal, which was to involve parents in the research process of co-developing KT tools and informing research strategies. The evaluation involved: a baseline survey to understand parents’ motivations for joining the group; a mid-year focus group to identify any problems or challenges with the groups’ operations; and a year-end survey with optional one-on-one interviews to understand parents’ experiences with the group in an effort to identify ways we could improve the group’s operations.

We have previously reported on development of the P-PAG and evaluation from the first year of operating the group (2016) [6]. We did not conduct an evaluation during 2017 due to staffing turn-over. In this paper, we present the results of year-end evaluations for four years, from 2018 to 2021. P-PAG terms took place from September to June; we have referred to each term by the year the term started (i.e., 2018 refers to the 2018–19 term). Further, we discuss our experiences coordinating the group and the changes we have made in response to member feedback. Our experience and the evaluation described in this paper are unique as we have been maintaining the group over many years. We trust that our experiences will be of value to other researchers and research-focused organizations interested in engaging patients or patient advocates in their work.

## Methods

The purpose of the evaluation plan was to learn about parents’ experiences as P-PAG members, and to document and improve P-PAG processes with the aim of providing a meaningful experience for members while meeting the goals of our research program. Key objectives of the evaluation are outlined in Table 1. We applied a mixed methods approach, collecting both quantitative (survey) and qualitative (survey and interview) data. Results from four year-end surveys and interviews from 2018 to 2021 are presented in this manuscript.

**Table 1:** P-PAG evaluation objectives

· Understand parent expectations and motivations for participating in the P-PAG, and how they may change over the course of participation · Identify factors that make participation in the P-PAG meaningful and worthwhile for parents · Identify facilitators and barriers to participation in the P-PAG · Determine if participation in the P-PAG furthers the KT goals of the research program · Provide a mechanism for feedback from the P-PAG participants to identify concerns/challenges to participating and ensure they are addressed	

### Ethics approval

This study was approved by the University of Alberta Health Research Ethics Board (Pro00066847) and all participants provided informed consent prior to data collection.

### Evaluation components

#### Year-end survey

The year-end survey was sent to P-PAG members towards the end of each term. The survey asked parents about their experience being a P-PAG member, as well as their views on its management and interactions with the research team. Parents were also provided with the opportunity to add questions, comments, and concerns at the end of the survey via an open-ended/free-text response box.

All P-PAG members were eligible and invited to participate. The process and purpose for the evaluation was described by the research team at P-PAG meetings prior to being sent to members via email. The email explained the purpose of the evaluation and contained a link to the survey, information letter, and consent instructions. Consent was implied by completion and submission of the survey. No identifying information was collected, and all responses were anonymous. The research team communicated that participation in the survey was voluntary and was not required as part of group membership, nor would decision to participate affect their involvement in the P-PAG.

The survey was administered using SimpleSurvey [9], a secure online software program. Survey questions regarding outcomes of membership (n = 9), experience with the P-PAG (n = 16) and interactions with the research team (n = 7) were assessed using a 7-point Likert scale, ranging from “1 = Strongly Disagree” to “7 = Strongly Agree”. Questions about participant confidence using research and supporting their child’s health (n = 8) used a scale from 1 to 10 (1 = “Not at all” confident; 10 = “Extremely” confident). Questions assessing perceptions of P-PAG management (n = 7) used a 5-point Likert scale, ranging from “1 = Ideal” to “5 = Unacceptable*”*. The survey concluded with several open-ended response questions.

#### Year-end interviews

P-PAG members who completed the survey were invited to participate in one-on-one semi-structured interviews to discuss their experience in the group and to contextualize the survey findings. Interviews were scheduled via email and conducted either in person, via telephone, or over Zoom [10], at a time and setting convenient for the participant. An interview guide was developed by the research team that included parallel questions to those used in the survey. Research staff trained in qualitative data collection conducted interviews. Interviews were audio recorded either through Zoom’s local recording feature, using a digital recorder, or via a secure phone audio recording system. Recordings were transcribed verbatim and de-identified by a research team member in the first year, and by a professional third-party transcriptionist service (Simply Transcription [11]) from 2019 onwards. After survey and interview results were analyzed, we presented a summary of the findings to the parents at a regular P-PAG meeting and discussed potential implications (e.g., changes to group operations going forward).

### Data analysis

#### Survey data analysis

Survey data were managed and analyzed in Microsoft Excel [12] (numerical responses) and Microsoft Word [13] (open-ended responses). Data were analyzed by term using descriptive statistics (i.e., frequencies, percentages). Responses to questions using the 7-point Likert scale were grouped into three categories: (1) “Strongly Agree” and “Agree”; (2) “Somewhat Agree”, “Neutral”, “Somewhat Disagree”; and (3) “Disagree” or “Strongly Disagree” (responses by original categories are available in Additional file 1). Responses to questions using the 5-point Likert scale were grouped into three categories: (1) “Ideal” or “Good”; (2) “Neutral”; and (3) “Poor” or “Unacceptable”. Some parents were members of the group for multiple years; hence, statistical comparisons across years were not possible as responses were not independent. Open-ended text responses were analyzed by conventional content analysis [14]. Survey comments were read repeatedly, and codes were identified that captured key concepts. Notes of first impressions and initial analysis were documented. Codes were then organized into categories. Preconceived categories were not used in the analysis, however inductively derived categories and illustrations (quotes) were presented alongside the structured quantitative survey sections (e.g., P-PAG management; researcher interaction, etc.).

#### Interview data analysis

Data management and thematic analysis were facilitated using NVivo 12 Software [15]. The thematic analysis process followed that as outlined by Braun and Clarke [16, 17] and entailed familiarization with the data, initial coding, grouping similar codes together, and development of themes. Transcripts were read several times, organized based on area of interest (e.g., P-PAG management, researcher interaction), coded, and analyzed to identify common themes across the interviews.

An inductive, line-by-line approach was employed during the coding process. Data were coded, sorted, and labeled based on the themes that developed throughout the analysis. Analytic rigour and trust were promoted through continual communication with the research team. Interview recordings and detailed field notes promoted confirmability of the findings. Field notes describing the researcher’s initial impressions were kept to promote reflexivity, allowing for acknowledgment of bias, transferability, dependability, credibility, and an audit trail [18].

#### Mixed methods integration

We used a convergent mixed methods design, in which quantitative and qualitative data collection occurred within similar timeframes [19]. Analysis occurred following the conclusion of all data collection, with survey and interview data analyzed separately first. Quantitative and qualitative data were then integrated through merging (i.e., databases were brought together to be analyzed and compared) and interpreted contiguously, an approach in which qualitative and quantitative findings are presented in different sections of a single report [19]. The coherence of the qualitative and quantitative findings was assessed for confirmation, expansion, and discordance, and interpreted using joint displays.

## Results

### Group membership and evaluation participation

The size of the P-PAG varied over time, ranging from 16 members (2019) to 26 members (2018). The number of new and returning members also varied over the terms. When possible (with member’s agreement), exit interviews were conducted when members elected not to return for the following year, with the most common reason being competing priorities. Year-end evaluation response rates were: 15/26 (58%) with 5 interviews for 2018–19, 12/16 (75%) with 2 interviews for 2019–20, 15/24 (63%) with no interviews for 2020–21, and 8/23 (35%) with 2 interviews for 2021–22.

### Experience with P-PAG

#### Participation in the P-PAG

A high percentage of parents (87–100%) in each of the four years agreed or strongly agreed that they understood the purpose of the P-PAG and their role with the P-PAG. The percentage of parents reporting they had the supports they needed to participate in P-PAG increased from 67% in 2018 to 92–100% in subsequent years.

### P-PAG discussions and communication

**Fig. 2:**
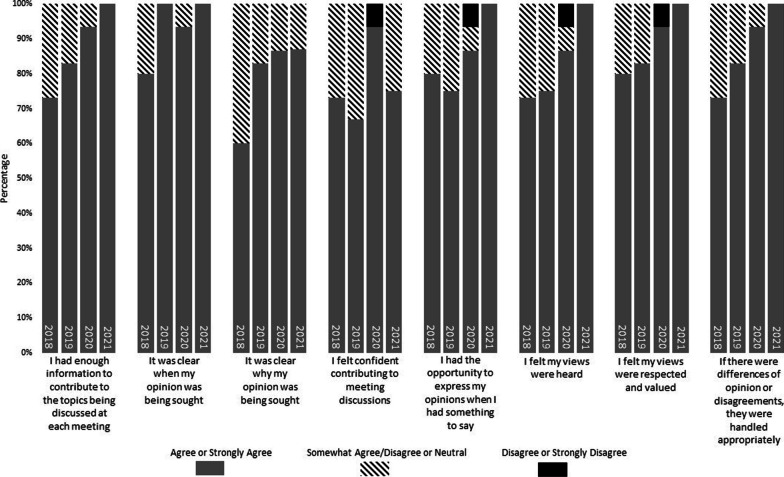
Results from year-end survey: P-PAG discussions and communication

Parents’ views on the discussions and communication within P-PAG meetings (Fig. 2) generally improved over time (i.e., percentage of parents selecting “agree” or “strongly agree” increased over time for most items). This included reporting that they had enough information to contribute to the topics being discussed at meetings (an increase from 73% to 100% of parents who agreed/strongly agreed); knew when (80% to 100%) and why (60% to 87%) their opinions were being sought; felt their views were heard (73% to 100%), respected and valued (80% to 100%); and differences of opinion or disagreements were handled appropriately (73% to 100%). There was some variability over the years in the percentage of parents reporting they felt confident contributing to meeting discussions (ranging from 67% to 93% who agreed/strongly agreed) and had the opportunity to express their opinions (ranging from 75% to 100% who agreed/strongly agreed).

### Impact of the P-PAG and the research program

**Fig. 3:**
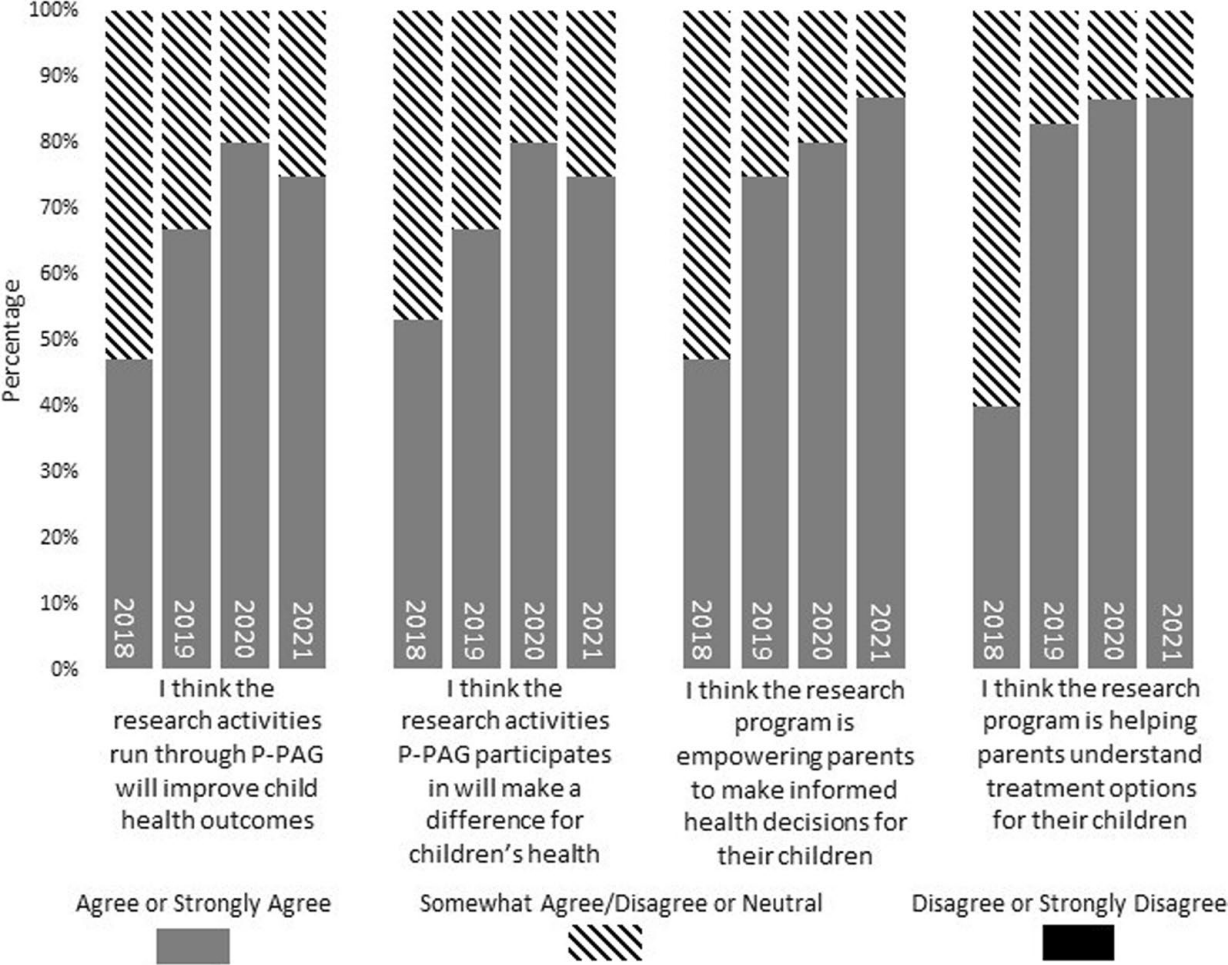
Results from year-end survey: Impact of the P-PAG and the research program

In the years following 2018, there was an increase in the percentage of parents who agreed or strongly agreed that the research activities run through P-PAG will improve child health outcomes (47% in 2018, 67% to 80% in subsequent years) and make a difference for children’s health (53% in 2018, 67% to 80% in subsequent years). Also showing an increase over time was the number of parents who agreed/strongly agreed that the research program is empowering parents to make informed health decisions for their children (47% in 2018, 87% in 2021), and the research program is helping parents understand treatment options for their children (40% in 2018, 87% in 2020 and 2021). These results are presented in Fig. 3.

### Management of P-PAG

**Fig. 4:**
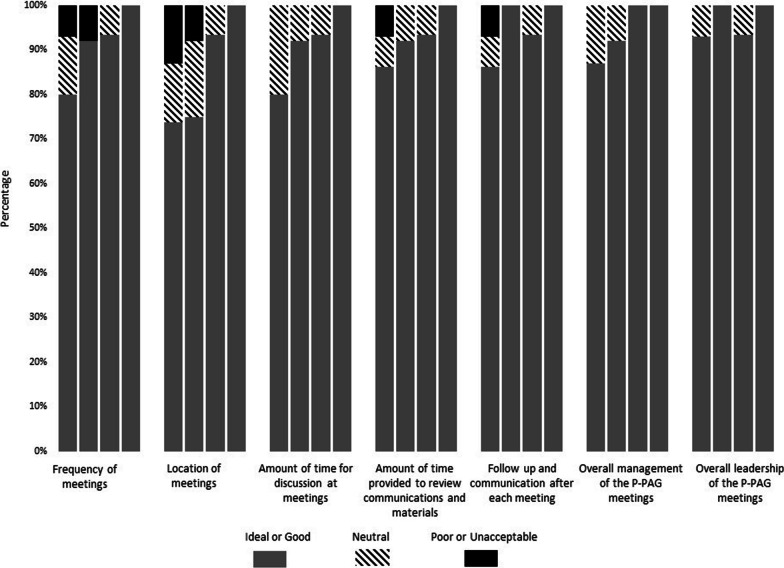
Results from year-end survey: Management of P-PAG

Figure 4 presents parents’ views regarding the management of P-PAG. Each item was highly rated across the years (73% to 100% of members rated items ideal or good). The highest rated items were overall management of the P-PAG (rated ideal or good by 87% to 100% of members) and overall leadership of the P-PAG (rated ideal or good by 93% to 100% of members). Over the years, there was an increase in the percentage of parents rating the following aspects of P-PAG management as ideal or good: frequency of meetings (80% to 100%), amount of time for discussion at meetings (80% to 100%), and amount of time provided to review communications and materials (87% to 100%). Location of meetings was rated most highly in 2020 (93% ideal or good) and 2021 (100% ideal or good); meetings had previously been in person but were held virtually during these years due to the COVID-19 pandemic.

### Confidence regarding research, KT, and supporting their child’s health

#### Confidence regarding research and KT

**Fig. 5:**
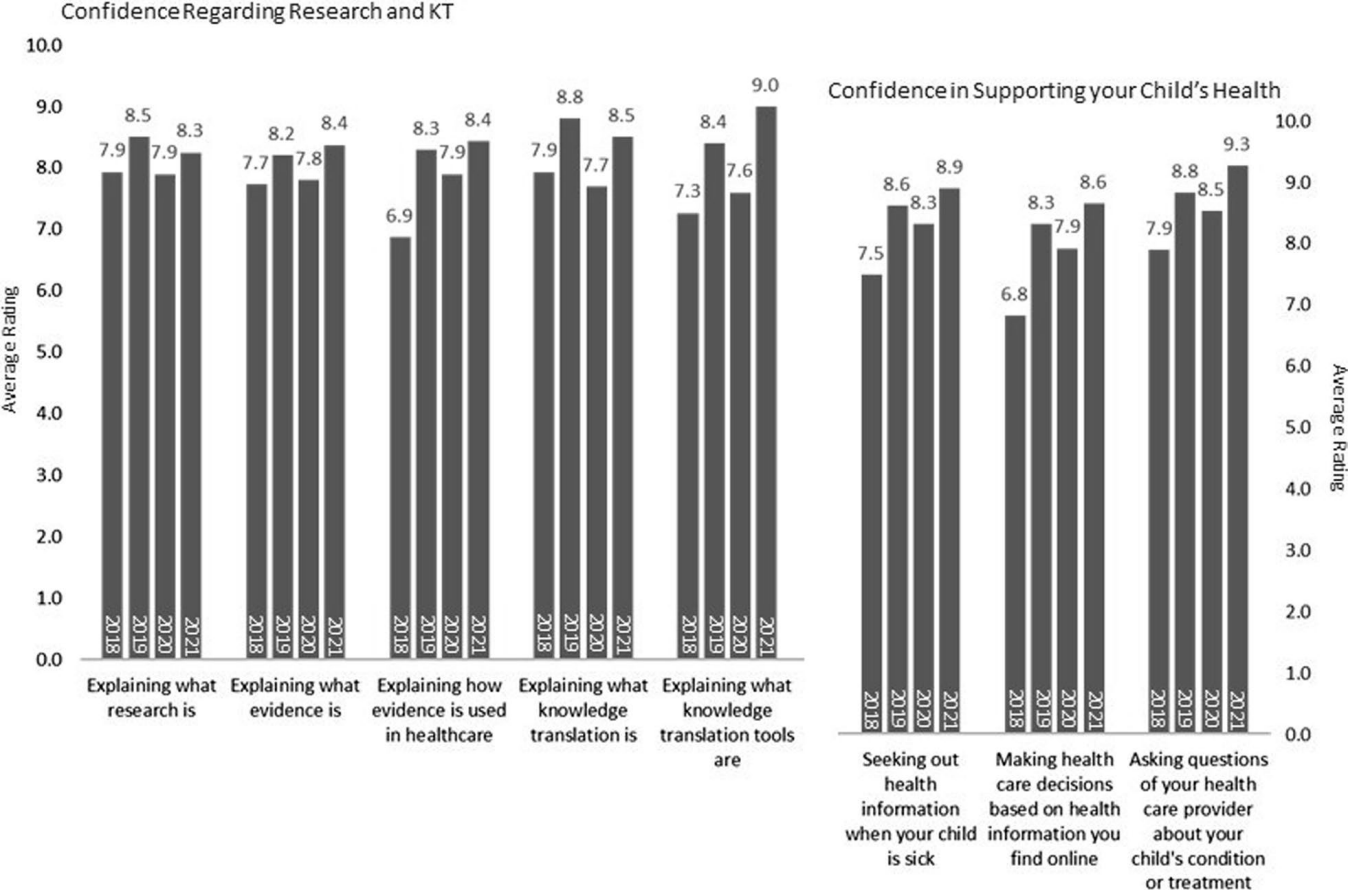
Results from year-end survey: Confidence regarding research, KT, and supporting your child’s health

These results are presented in Fig. 5. In the years following 2018, there was an increase in parents’ reported confidence in explaining both how evidence is used in healthcare (6.9 in 2019 to 8.4 in 2021; scale of 0–10) and what KT tools are (7.3 to 9.0). Confidence in explaining what research (7.9 to 8.3), evidence (7.7 to 8.4), and KT (7.3 to 9.0) showed a general improvement but also variability over the years.

### Confidence in supporting their child’s health

Although not reflective of the purpose or objectives of the P-PAG, these questions assessed secondary outcomes related to parents’ involvement with the group, including parents’ actions and learnings. Parents’ reported confidence increased after 2018 regarding seeking out health information when their child is sick (from 7.5 in 2018, then 7.9 to 8.9 in subsequent terms) and making health care decisions based on health information they find online (from 6.8 in 2018, then 7.6 to 8.9 in subsequent terms). Confidence in asking questions of their health care provider about their child’s condition or treatment varied over time (ranging from 7.4 to 9.3).

### Researcher interaction

Across all years, most parents agreed or strongly agreed that the research team was responsive to feedback (73% to 100%) and approachable (87% to 100%), the team’s contributions to discussions were appropriate (80% to 100%), and they appreciated having the lead researchers at the meetings (73% to 87%). A minority of parents (25% to 37%) agreed or strongly agreed that they would have liked to hear more from the lead researchers.

### Outcomes of P-PAG membership

**Fig. 6:**
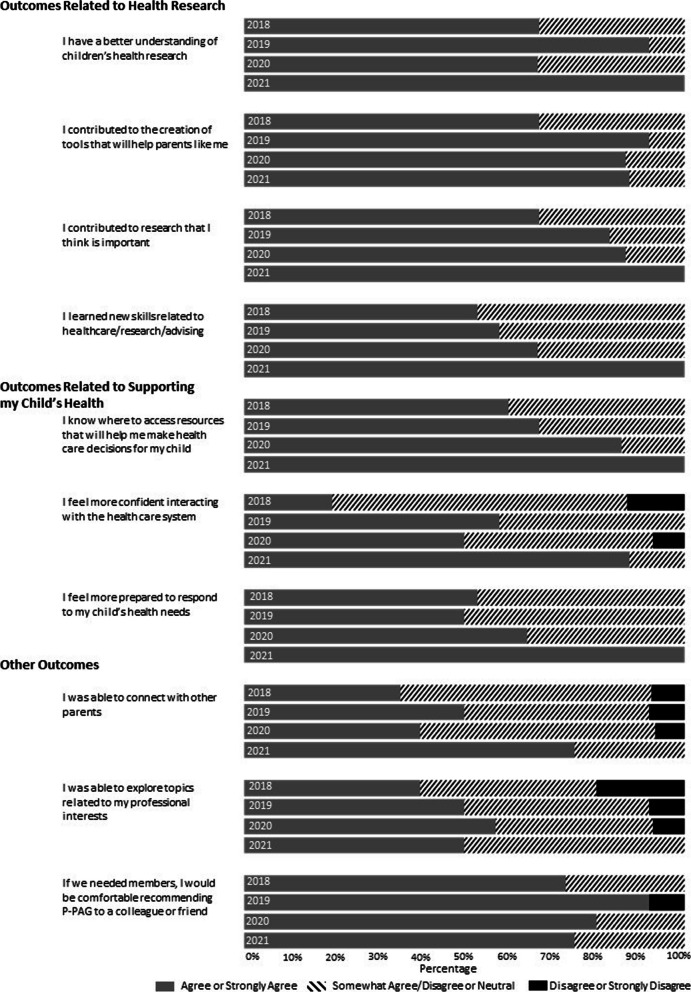
Results from year-end survey: outcomes of P-PAG membership

The results related to outcomes of P-PAG membership are presented in Fig. 6. While there was variability in responses across the years, the majority of parents (over 75%) agreed or strongly agreed with all items in the most recent year.

### Outcomes related to health research

The majority of parents in each year agreed or strongly agreed that they have a better understanding of children’s health research (67% to 100%) and learned new skills related to healthcare/research/advising (52% to 100%). Most parents agreed or strongly agreed that they contributed to the creation of tools that will help other parents (67% to 92%) and research they considered important (67% to 100%).

### Outcomes related to supporting their child’s health

Most parents agreed or strongly agreed they know where to access resources to help make healthcare decisions for their child (60% to 100%), and they feel more prepared to respond to their child’s health needs (53% to 100%). Parents’ reported confidence in interacting with the healthcare system varied over time, ranging from 20 to 88% of parents who agreed or strongly agreed.

### Other outcomes

There was an increase over the years in the percentage of parents who agreed or strongly agreed that they were able to connect with other parents (33% in 2018, reaching 75% in 2021). There was also an increase in the number of parents who agreed or strongly agreed that they were able to explore topics related to their professional interests (from 53% in 2018 to 100% in 2021). The percentage of parents who agreed or strongly agreed they would be comfortable recommending P-PAG to a colleague/friend varied over time, ranging from 73% in 2018 to 92% in 2019.

### Qualitative results

Themes from interviews and open-ended survey questions are presented in Table 2. Identified themes included the importance of certain elements of the P-PAG (e.g., supports in place to facilitate participation, contributions to child health research), parents’ positive views of different aspects of the group’s functioning (e.g., organization of meetings, safe space for discussion, interaction with lead researchers), and several potential areas for improvement (e.g., clarifying and making more explicit the role and purpose of the advisory group, providing more context or background for discussions). Themes have been presented to parallel the survey questions where possible.

**Table 2:** Qualitative results

Evaluation question	Topic	Theme	Illustrative quotes
Experience with P-PAG	Participation in the P-PAG	Importance of clarifying the role and purpose of the advisory group in the early stages of group development	“I would probably suggest to try to make the…context or the purpose more clear at the beginning” (IP1_2018-19) “Perhaps it would be best if—if one were starting from square one, to anticipate that…and to explain to the participants ah… ‘Here’s the extent, and here’s the limit of your participation.’” (IP2_2018-19)
		Importance of supports in place to facilitate participation	“actually reimbursement of like, travel expenses and providing hospitality goes a long way” (IP3_2018-19) “Make it convenient, in terms of location and parking, timing—all of that stuff” (IP4_2018-19)
	Discussions and communication	Parents valued discussion re: their views on the P-PAG’s role, purpose, and vision	“I like the sessions where we were kind of um, strategizing about um… where the group could make an impact, or where it wants to make contributions and so on” (IP4_2018-19) “I thought it was really interesting that the P-PAG was flexible and started to make time and build it into um, the monthly meetings for us to sort of um, step back and look at what the group could and would accomplish if we had the opportunity” (IP3_2018-19)
		Positive view of P-PAG discussions and communication within group	A survey respondent in 2018–19 stated what they enjoyed most about the P-PAG was the “respectful and inclusive atmosphere; [and] sincerity of researchers in incorporating feedback” (SP1_2018-19) When asked in the 2020–21 survey if there was anything they would change, a parent stated: “I wish members participated more equally, but I think there is ample opportunity for folks to weigh in” (SP1_2021–22)
		Importance of context/background for discussions	“I think sometimes it would be good if they [the researchers]… piped up a little bit more at times…and maybe provide some…feedback on…additional background” (IP04_2018-19) “Sometimes items need more context prior to jumping into discussions” (SP3_2018-19)
	Impact of the P-PAG and overarching research program	Parents want to hear and learn more about the impact of P-PAG activities	“It would be really cool to hear that you know, we talked about this being at different health centres, and it’s gone to now like three health centres, or um… like there was ah, 811 picked this up. They put it on the web site. I love seeing things sort of implemented at that level” (IP3_2018-19) Another parent suggested “seeing what the follow up of our work looks like, as it gets implemented. Um… you know, so that we have a sense of the impact that—that this—this stuff could have” (IP4_2018-19)
Management of P-PAG		Positive view of how P-PAG was managed	“It was organized and felt like a safe space to discuss topics” (SP4_2021-22) “I feel that P-PAG is well-managed and very organized” (SP9_2020-21)
		Positive view of the move to virtual meetings	“Everyone in the world was forced to move to remote and a Zoom kind of experience. That has definitely been on the positive side, it’s made it a lot easier to participate” (IP1_2019-20) “This format works for me because had this Covid not happened, I would never have been able to participate” (IP2_2021-22, member from outside of Edmonton)
		Diversity of membership as potential area for improvement	“So many people had professional qualifications…or were working on them in the health care field” (IP2_2018-19) “I still think we need to endeavor to find a more variety of people representation as far as diversity” (IP2_2021-22)
Confidence Regarding Research, KT, and Supporting their Child’s Health	Confidence supporting child’s health		“My son had an acute condition over the time that I was in P-PAG…I feel like it did actually empower me in going through the health care system to know um, what was happening when people were providing me with resources such as clinical care sheets and things. I sort of had—although I had a general sense before, I felt a little bit um, stronger in that” (IP3_2018-19)
Researcher Interaction		Some parents found volume/nature of researcher interaction appropriate	“It would have been easy for them to take over the meeting, but they have…they were very scrupulous in—in avoiding that” (IP2_2018-19) “I think it’s neat that they didn’t talk a lot. And when they did talk, it was often, you know, very informally and sort of like hearing from a friend, rather than an authority” (IP3_2018-19)
		Some parents wanted to hear more from researchers	Regarding wanting more input from researchers at meetings, parents wanted to hear more about the context of the group’s activities; one parent suggested researchers “provide some of the background and some of—the ways the tools were developed, and where the information came from” (IP5_2018-19) “I think sometimes it would be good if they… piped up a little bit more at times…and maybe provide some…feedback on ideas that might be kicking around the table, or… um, additional background on why an idea might be feasible, or why it might not be” (IP4_2018-19)
Outcomes of P-PAG membership	Outcomes related to health research	Parents noted an increased awareness of research	“I just enjoy having some exposure to the world of evidence and child evidence, and the world of research” (IP1_2018-19)
		Parents expressed wanting more learning opportunities	A parent suggested “an in-service every now and then where…they present information on topics relevant to the group. So there’s a little bit of group learning that takes place” (IP4_2018-19) A parent suggested: “possibly add more presentations by researchers that are solely for information for parents” (SP6_2020-21)
		Parents valued the contribution to child health research they made through the P-PAG	“I really enjoyed knowing that I was contributing to upcoming information for parents and families” (PX_2020-21) A parent most enjoyed “contributing to health research that is accessible to parents” (SP2_2019-20)
	Supporting child’s health		A parent stated what they enjoyed most about the P-PAG was “Learning about different topics I will encounter in my own parenting journey…so I can cater my parenting to my learning!” (SP3_2020-21)
	Other outcomes	Parents appreciated and valued connections with other parents in the P-PAG	“I thought it was really satisfying to meet with the other parents” (IP3_2018-19) “I’ve always enjoyed the…interaction among…the members of—of PPAG” (IP2_218-19) A parent enjoyed most about the P-PAG “The opportunity to discuss health topics with other parents” (SPX2_2019-20)

*IP* interview participant, *SP* survey participant

## Discussion

Developing, sustaining, and evaluating a parent advisory group for our research program over many years has provided important insights into parent (as patient proxies) involvement in health research. There is growing interest in patient engagement in health research, yet there is a paucity of studies evaluating advisory groups with this purpose. Specifically, there is little empirical evidence about patients’ experiences, whether they are engaged in a meaningful way, and whether their involvement meets their own and the researchers’ expectations. It is critical to build an evidence base regarding what works (and what does not work) in sustaining patient engagement over time [20]. Findings from this four-year evaluation have highlighted the importance of a rigorous and ongoing evaluation plan, key strategies and requirements for sustaining the group over time, and the direct and indirect outputs from patient involvement in research.

### Evaluation and response to feedback

Results of the year-end surveys generally demonstrated improvements over time. Findings from ongoing evaluations were carefully considered by the research team, which prompted targeted efforts to make changes and improve P-PAG operations. Specific changes in response to parent feedback included frequency and location of meetings, regular communications about the purpose of the group, offering sufficient context and time for individual discussion items, and providing feedback to parents about how their input was used. This intentional, iterative approach has been essential for fostering meaningful engagement. Notably, we saw 100% agreement with items related to the management of the P-PAG in the most recent year. Responsiveness of the research team (and how parents valued this) was a major theme within the qualitative data. The importance of assessing the effectiveness of patient engagement approaches and being responsive to feedback through adjustments and improvement has been previously highlighted in the literature as critical to achieve engagement [21–23].

Beyond communications and management of the P-PAG, our evaluation gathered parents' perspectives on the impact the group is having on the research program’s intended goals (i.e., co-development and dissemination of KT tools). While parent responses suggested they perceived an impact in terms of proximal outcomes (e.g., they contributed to tools that would help other parents and research they felt was important), there was lower agreement from parents that the research activities would improve child health outcomes or make a difference for children’s health. This was reinforced through the qualitative results indicating that parents wanted to hear more about the downstream impact of the research group’s activities. This presented a challenge for the researchers as we are not able to capture data on more distal outcomes, but it was important to understand as this mismatch could lead to lower engagement and retention of members. It also underscored the importance of clearly articulating the purpose of the group, the role the researchers envisioned for members, and where the researchers’ work fits into the larger healthcare and health research ecosystem (e.g., how and when results from individual research and KT initiatives are implemented in the health system).

### Sustaining P-PAG

Most of the patient engagement literature involves findings from engagement efforts specific to one research project or initiative. The P-PAG is unique in that members have informed numerous projects and informed a variety of KT strategies within a program of research over an extended period of time. To our knowledge, the challenges related to long-term sustainability have not been explored in the literature. In addition to the findings from our evaluation plan, many operational learnings took place over the years. First and foremost, we realized that significant resources are required to maintain an advisory group, a longstanding message in the patient engagement literature [21, 22, 24]. Maintaining a group of this nature necessitates funding for dedicated and highly skilled staff to support the ongoing operations of the group (e.g., communications, meeting planning and facilitation, recruitment, onboarding, etc.), as well as evaluation efforts to regularly seek members’ feedback, including strengths and potential areas of improvement. We experienced a change in coordinators over the years often due to the contractual and short-term nature of funding for the position; this presented challenges and underscored the critical importance of organizational skills including detailed record keeping. In addition to staff costs, funding must be available to compensate parents for their time as well as the costs of attending meetings (e.g., travel, parking, childcare).

Due to the ongoing nature of the group, we experienced regular turn-over of membership at the end of each year. This necessitated time and effort for regular recruitment and orientation. Further, we had a regular mix of returning members and new members, which created a challenge to ensure new members felt engaged and comfortable participating, alongside those with more experience. We also maintained a relatively large group (16 to 26 members) as we saw early on that parents were not able to attend every meeting, and we wanted to have at least 8 to 10 members at each meeting to allow for meaningful discussion and varied input. Further, we continuously sought to increase diversity in the group (e.g., members with different parenting roles; parents with different ethnic, educational, and socio-economic demographics, etc.). Notably, recommendations for the size of patient advisory groups have not been clearly outlined in the literature [25]; this issue merits more attention and likely varies with the purpose and goals of a given group. Furthermore, guidance on diversity and mechanisms to achieve diversity within health research engagement are lacking. Maintaining diversity in group membership was a challenge identified during the first year of the P-PAG [6].

An important observation in sustaining an advisory group is that there may be significant changes over time and there is a need to be flexible, responsive to feedback, and open to change. With the P-PAG, we experienced changes in the size and membership of the group, as well as changes in the nature of parent participation (i.e., different projects and types of input sought). While we started meeting in-person, we had to move to virtual meetings during the COVID-19 pandemic. While this was initially considered less optimal, we heard from parents that it allowed for: greater participation on their part (e.g., no need for childcare, less time and cost to travel to meetings); participation from a more diverse geographic area; and more equitable participation (i.e., everyone online while previously we had a mix of parents attending in-person and online).

### Outputs of the P-PAG

The P-PAG has been critical to advancing our research program and the outputs have been extensive. P-PAG members have participated in the co-development of over 40 KT tools for parents, provided input on methods and recruitment strategies for numerous research projects [26–40] and informed dissemination strategies. Other researchers as well as trainees (undergraduate and graduate students, post-doctoral fellows) have accessed the group for input on a range of projects and initiatives. From a researcher’s standpoint the contributions of the P-PAG have been immense and invaluable in terms of direct outputs. The evaluation also allowed us to assess indirect outputs of participation in the P-PAG, such as parents learning about research and KT, and gaining confidence in managing their children’s health and interacting with the health system. We did not explore the mechanisms for these changes; however, we expect that increased exposure through regular discussions about research and KT as well as contributing to the KT tools likely impacted parents’ knowledge and confidence. Moreover, we made intentional efforts over the years to regularly discuss our research processes [6] and involved the parents in a number of KT and dissemination projects and activities [28, 41]. Over the years, parent members have participated in presentations and workshops at scientific meetings and conferences, and have been co-authors on scientific publications in peer-reviewed journals. While these indirect outputs are positive and encouraging, P-PAG members have raised questions about when their input may no longer reflect an “average” parent. This is a critical area to explore within the field of patient involvement in research.

### Strengths and limitations

We have conducted a rigorous evaluation using multiple methods over multiple years of running a parent advisory group which has resulted in many rich findings and allowed us to effectively adapt the group with a goal of optimizing parent engagement. The aim of our evaluations was specific to parents’ involvement and engagement; we did not intend to assess the impact of their contributions on research outcomes. A potential limitation of this evaluation is participation bias; the views of those members who opted to participate in the year-end surveys and interviews may not be reflective of the full group. Further, we don’t know whether survey participants were new or returning members. As well, participation rates in the survey decreased over time. Members who chose to participate in the evaluations may have been those who had the most positive view of their experience with the P-PAG; alternatively, we heard from parents that they sometimes chose not to participate in the evaluation if they felt there was nothing to change or improve upon. A related limitation is the sample size which could be considered small for some of the year-end surveys and interviews; however, we had a fixed target sample and, while we encouraged parents to complete the evaluations we also stressed that their participation was voluntary and would not impact membership in the group. Interpreting the findings and comparing data across the years was complicated by changes in group membership. There has also been overlap in membership over time (i.e., some parents participated in the P-PAG for multiple years). However, parents who have been with the group over multiple years (including parent co-authors) have noticed changes and responsiveness from the researchers to continuously improve operations and engagement. We did not conduct interviews with or seek feedback from other members of the research team (e.g., staff who coordinated P-PAG) or feedback about specific roles that parent members may have had (e.g., chair, presenter, author).

An additional limitation is that “meaningful engagement” is not well defined in the POR literature and there are no validated instruments to measure this construct. Consistent with others [24], we found that parents had varied perceptions of what contributed to meaningful engagement. For example, we heard that parents valued seeing their input incorporated into amended versions of KT tools; however, they indicated less agreement with more downstream (impact on child health outcomes) or peripheral (opportunity to connect with other parents) outputs.

### Future directions

When the P-PAG was first established in 2016, not much was known about how to develop patient or parent advisory groups or meaningfully engage patients and the public in health research. Interest and research in patient engagement (and recognition of its importance) has increased considerably over the past several years, including the development of frameworks for the conduct and evaluation of patient engagement [8, 42–44]. Our experience with the P-PAG leads us to agree with identified research priorities [8, 44] including the incorporation of patient engagement evaluations into broader research evaluations and the significant role of evaluation in improving the conduct and demonstrating the value of patient engagement efforts. We would encourage researchers to incorporate regular evaluations of their patient engagement efforts, carefully aligning the evaluations with the purpose and activities specific to their context. To our knowledge there are no validated tools to assess engagement in advisory groups; in light of this, we would recommend building evaluation tools with patient/parent input (e.g., for face validity) and pilot testing the tools to ensure they capture the type of feedback intended. Researchers may also consider the Patient Engagement Evaluation Tool (PEET) which was validated to assess patient engagement in development of clinical practice guidelines [45]. Further, funding agencies who encourage patient engagement should support costs and infrastructure to assess related outcomes.

Evaluating the impact of patient involvement in research is associated with various challenges but should be a priority for the field [2]. There is an imperative for researchers to measure what is feasible in POR, including value to patients/parents (e.g., increased knowledge of research), building towards a framework for the measurement of POR impacts on health outcomes [2]. We hope this research contributes to this collective effort.

## Conclusions

The P-PAG has been critical to advancing our research program and the outputs have been extensive; moreover, we feel that our outputs have greater relevance to parents because of the P-PAG’s input. Our multi-method evaluation over multiple years has provided numerous insights into the parents’ experiences being part of a group that supports health research. Our findings have provided an understanding around strategies and requirements for sustaining the group and engaging parents (i.e., dedicated and highly skilled staff, adequate resources, clear purpose, effective communication, regular feedback on how parent input is used), as well as the value of the group in terms of direct and indirect outputs of parent involvement. Further, our evaluation underscores the importance of seeking regular feedback from group members and continuously adapting to improve their experience and engagement. Further research is needed to evaluate the impact of patient/parent involvement in research and to define and develop tools to assess “meaningful engagement.” To manage expectations and optimize engagement, we found it was critical to clearly articulate the purpose of the group, the role the researchers envisioned for members, and where the researchers’ work fits into the larger healthcare and health research ecosystem.

### Supplementary Information

**Additional file 1.** P-PAG Year-end Survey Responses 2018-2022. Response rates and responses to year-end survey questions grouped by original categories from 2018 to 2022.

## Data Availability

The study materials (year-end survey, interview guide), datasets used and/or analyzed during the current study are available from the corresponding author on reasonable request.

## Abbreviations

KT: Knowledge translation
POR: Patient-oriented research
P-PAG: Pediatric parent advisory group
CIHR: Canadian institutes of health research
